# Quantum chemical calculation dataset for representative protein folds by the fragment molecular orbital method

**DOI:** 10.1038/s41597-024-03999-2

**Published:** 2024-10-23

**Authors:** Daisuke Takaya, Shu Ohno, Toma Miyagishi, Sota Tanaka, Koji Okuwaki, Chiduru Watanabe, Koichiro Kato, Yu-Shi Tian, Kaori Fukuzawa

**Affiliations:** 1 Graduate School of Pharmaceutical Sciences, Osaka University, 1-6 Yamadaoka, Suita, Osaka 565-0871 Japan; 2 Center for Biosystems Dynamics Research, RIKEN, 1-7-22 Suehiro-cho, Tsurumi-ku, Yokohama, Kanagawa 230-0045 Japan; 3 Department of Applied Chemistry, Kyushu University, 744 Motooka, Nishi-ku, Fukuoka 819-0395 Japan

**Keywords:** Scientific data, Biophysical chemistry, Data publication and archiving, Cheminformatics, Quantum mechanics

## Abstract

The function of a biomacromolecule is not only determined by its three-dimensional structure but also by its electronic state. Quantum chemical calculations are promising non-empirical methods available for determining the electronic state of a given structure. In this study, we used the fragment molecular orbital (FMO) method, which applies to biopolymers such as proteins, to provide physicochemical property values on representative structures in the SCOP2 database of protein families, a subset of the Protein Data Bank. Our dataset was constructed by over 5,000 protein structures, including over 200 million inter-fragment interaction energies (IFIEs) and their energy components obtained by pair interaction energy decomposition analysis (PIEDA) using FMO-MP2/6-31 G*. Moreover, three basis sets, 6-31 G*, 6-31 G**, and cc-pVDZ, were used for the FMO calculations of each structure, making it possible to compare the energies obtained with different basis functions for the same fragment pair. The total data size is approximately 6.7 GB. Our dataset will be useful for functional analyses and machine learning based on the physicochemical property values of proteins.

## Background & Summary

The three-dimensional structures of biological macromolecules such as proteins and nucleic acids are crucial for understanding their functions. These structures can be determined experimentally using X-ray crystallography, nuclear magnetic resonance spectroscopy, and cryo-electron microscopy. The results of this study make more than 200,000 structures available from the Protein Data Bank (PDB) on the websites of the wwPDB group members^1–3^. Recently, AlphaFold2^4^ has made it possible to generate accurate protein model structures even in the absence of experimental information. Uniprot^5^ provides a database of AlphaFold2 model structures, called the AlphaFold Protein Structure Database (AlphaFold DB)^6^. Because new insights obtained from such reliable structures are useful, the accumulation of computational data from simulations is expected to become increasingly important.

There are two major computational methodologies for biomacromolecules: molecular dynamics (MD) simulations^7^ for investigating dynamic behavior and quantum mechanical (QM) calculations for the precise electronic states. MD simulations are used to study loop flexibility, molecular conformation in solvents, and especially the interactions with ligand molecules. Although MD simulations account for the dynamic structural changes, they typically employ fixed charges. Biological macromolecules also perform their functions by forming specific atomic networks, including hydrogen bonds, ionic bonds, and nonpolar interactions, all of which involve the structure-dependent electronic state. QM is a promising non-empirical method though which the electronic state of a given molecular conformation can be determined. In general, the computational cost of QM calculations is approximately proportional to the fourth to sixth power of the number of basis functions; therefore, QM is mostly applied to small molecules. Several methods have been developed to overcome this limitation. QM/MM techniques such as ONIOM are hybrid approaches that logically partition molecules, enabling quantum chemical calculations in targeted regions and molecular force field calculations in others. Such methods have also been used to study chemical and enzymatic reactions^8^.

**Fig. 1:**
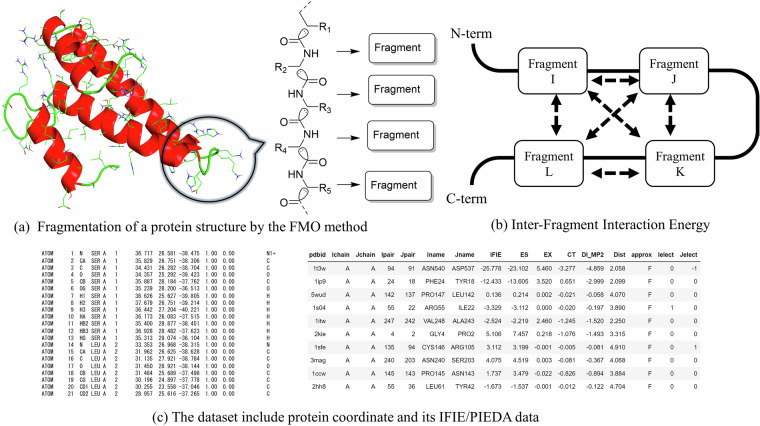
Summary of the dataset of QM-based energies of protein structures by the FMO method. (**a**) The structure of a protein can be divided into fragments based on amino acid units. (**b**) IFIE/PIEDA data are calculated based on interactions between fragments. (**c**) The dataset includes protein atomic coordinates and its IFIE/PIEDA energy data.

Currently, the fragment molecular orbital (FMO) method^9^ is the promising full-QM method applicable to biological macromolecules. The FMO method divides biological macromolecules such as proteins and nucleic acids into residual fragments and performs quantum chemical calculations (Fig. 1a). The FMO method has been implemented in software programs such as GAMESS^10–12^, and ABINIT-MP^13–15^ and is still under development.

The data obtained from the FMO method includes the inter-fragment interaction energy (IFIE also called pair interaction energy (PIE)), total energy, and atomic charge. IFIE/PIE has the advantage of describing residue-by-residue interactions and facilitating the energy interpretation of inter- and intramolecular interactions (Fig. 1b). Pair interaction energy decomposition analysis (PIEDA)^16^ is a method for analyzing the interaction between fragments that decomposes IFIE into electrostatic interaction (ES), exchange repulsion (EX), charge transfer with higher-order mixed-term interactions (CT + mix), and dispersion interaction (DI) components, and can be used to quantitatively determine which of these components is strongly involved in the binding between fragments. For example, hydrogen bonds, which frequently occur in the main and side chain interactions of amino acid residues, can be evaluated using in terms of the ES and CT + mix components. The DI component is particularly suitable for evaluating nonpolar interactions and contributes strongly to CH/π and π–π bonds^17–21^. Computational simulations for protein-ligand binding based on experimental structures have been reported^22,23^.

The IFIE and PIEDA in the FMO method have the following relationships. The total energy of a molecule can be calculated using the following equation^9^:

1 $${E}_{{\rm{total}}}\approx {\sum }_{I > J}^{N}\left({E}_{{IJ}}^{{\prime} }-{E}_{I}^{{\prime} }-{E}_{J}^{{\prime} }\right)+{\sum }_{I > J}^{N}{\rm{Tr}}\left({\triangle D}^{{IJ}}{V}^{{IJ}}\right)+{\sum }_{I > J}^{N}{E}_{I}^{{\prime} }$$

where ${E}_{{IJ}}^{{\prime} }$, ${E}_{J}^{{\prime} }$, and ${E}_{J}^{{\prime} }$ are the energies without environmental electrostatic potential between fragments *I* and *J*, fragment *I*, and fragment *J*, respectively, *N* is the number of fragments in the molecule, ${\triangle D}^{{IJ}}$ is the difference density matrix, and ${V}^{{\rm{IJ}}}$ is the electrostatic potential of the surrounding fragments. The IFIE is defined using the following equation:

2 $${\triangle E}_{{IJ}}=\left({E}_{{IJ}}^{{\prime} }-{E}_{I}^{{\prime} }-{E}_{J}^{{\prime} }\right)+{\rm{Tr}}\left({\triangle D}^{{IJ}}{V}^{{IJ}}\right)$$

The components of the PIEDA^16^ can be obtained from the following equation:

3 $${\triangle E}_{{IJ}}=\triangle {E}_{{IJ}}^{{\rm{ES}}}+\triangle {E}_{{IJ}}^{{\rm{EX}}}+\triangle {E}_{{IJ}}^{{\rm{CT}}+{\rm{mix}}}+\triangle {E}_{{IJ}}^{{\rm{DI}}}$$

where the IFIE is described by four types of energy terms.

As a quantum chemistry dataset, QM9 dataset is well known, which contains quantum chemical calculation values for molecular structures consisting of nine non-hydrogen atoms^24^. Our group also provides FMO calculation data from database, FMODB, containing the electronic states of biological macromolecules^25^. Currently, FMODB includes 37,450 entries constructed by the unique 7,783 PDB entries in 23 Jul 2024. Such datasets are used for machine learning applications, and all-electronic data on proteins are already being used for the construction of artificial intelligence platforms and other purposes^26^. The data registered in the FMODB depend on the interests of researchers. For example, there are many calculations for the Protein Kinase family (e.g., CDK2, p38 MAP, and Aurora), the nuclear receptor family (e.g., ERα and ERβ), the related proteins of SARS-CoV-2^27^, and apoproteins of X-ray crystal structure data^25,28^. The authors aim to make the FMO calculation results available for all structures deposited in the PDB for a wide range of applications of the FMO method. As of Sep 2024, there were more than 220,000 entries in the PDB; however, analyzing all entries is only possible if sufficient computing resources, such as supercomputers, could be used without restrictions. Because the convergency of FMO calculations depend on the atomic coordinate of proteins and can be unpredictable for individual proteins owing to variations in amino acid sequences, and crystallization conditions such as resolution, it is advisable to gather data on the convergence rate and distribution of FMO-based energies for representative structures before performing FMO calculations for all proteins in the PDB.

SCOP2, which is a database of protein folds, was selected as the dataset in this study to provide FMO calculation data for a wide range of proteins^29,30^. SCOP2 is a hierarchical classification of protein folds based on their structural and evolutionary relationships. It was derived from a subset of experimentally determined protein structures deposited in the PDB. The database is updated periodically to incorporate new families and structures. As of June 29, 2022, SCOP2 comprised 5,936 families. In this study, we present a comprehensive FMO computational dataset that encompasses all the experimentally characterized protein folds. This dataset, derived from protein structures associated with SCOP2 families, serves as a valuable resource for assessing the current capabilities of FMO methods, and enables researchers to readily access quantum chemistry data for folds of interest.

In the FMO method, as in any QM calculation, the judicious choice of calculation methods and basis sets is paramount for obtaining reliable and accurate results. The Hartree–Fock (HF) method is a fundamental ab initio quantum chemical method that utilizes the Hamiltonian operator and Slater determinant to approximate the ground state wave function of a molecular system. Although the STO-3G minimal basis set offers computational cost advantages, it requires at least double-zeta basis and the polarization functions in order to describe various interaction in biomolecules. In the context of FMO calculations, the MP2/6-31 G* level of theory (FMO-MP2/6-31 G*) is preferred because of the balance between accuracy and computational cost. This is because, in contrast to the HF method, the MP2 method (second order Møller–Plesset perturbation theory)^31–33^ can account for electron correlation, and the 6-31 G* basis set incorporates polarization functions for non-hydrogen atom polarization. The FMO-MP2/6-31 G* is frequently application in the study of relatively medium-sized organic compounds and the analysis of intermolecular interactions, including hydrogen bonding, CH/π^34^, and π–π interactions, between small molecules and proteins^35,36^. In addition, all of the data published in the FMODB uses this level of theory^25^. The validation of energy values derived from the FMO method, employing various combinations of calculation methods and basis sets, has been confined to a limited number of systems^37^. However, the recent development of supercomputers has enabled the use of higher levels of theory.

Basis functions are mathematical representations that approximate the spatial distribution of electrons within atomic orbitals. The characteristics of the basis sets used in this study are listed in Table 1. These functions are employed to express the molecular orbitals as linear combinations of atomic orbitals. In this study, we augmented the 6-31 G basis set by incorporating polarization functions for non-hydrogen atoms only and hydrogen atoms, denoted as 6-31 G* and 6-31 G**, respectively, thereby enhancing the accuracy of the electronic structure calculations. In addition, we used the correlation-consistent polarized valence double-zeta (cc-pVDZ) basis set, which was specifically designed to account for electron correlation effects. Consequently, our dataset now encompasses the FMO-MP2/6-31 G*, FM0-MP2/6-31 G**, and FMO-MP2/cc-pVDZ levels of theory. While MP2/6-31 G* only includes polarization functions (i.e., additional p-orbital functions) for non-hydrogen atoms, both MP2/cc-pVDZ and MP2/6-31 G** include them for hydrogen atoms. The cc-pVDZ basis set is distinguished by its utilization of Dunning-type functions and its design as a correlation-consistent basis set^38^. Since the formation of CH/π and π-π interactions through dispersion forces related to electronic correlations as well as hydrogen bonds contribute to protein folding, the use of either 6-31 G** or cc-pVDZ is considered necessary to properly evaluate the polarization of hydrogen atoms.

**Table 1:** Properties of the basis sets used in this study.

Basis set	Function type	Polarization		
		Non-hydrogen atoms	Hydrogen atoms	Correlation consistent
6-31 G*	Pople	✓		
6-31 G**	Pople	✓	✓	
cc-pVDZ	Dunning	✓	✓	✓

In summary, there is currently no quantum chemical dataset encompassing over 5000 protein structures classified into diverse families computed using multiple quantum chemical levels of theory. This dataset is not only instrumental for protein function and interaction analysis but is also anticipated to serve as training data for the development of machine learning models for protein charge prediction. Notably, providing energy values calculated using three distinct basis sets for the same fragment pairs facilitate the analysis of the effects of hydrogen atom polarization and electron correlation on intermolecular interactions.

## Methods

**Fig. 2:**
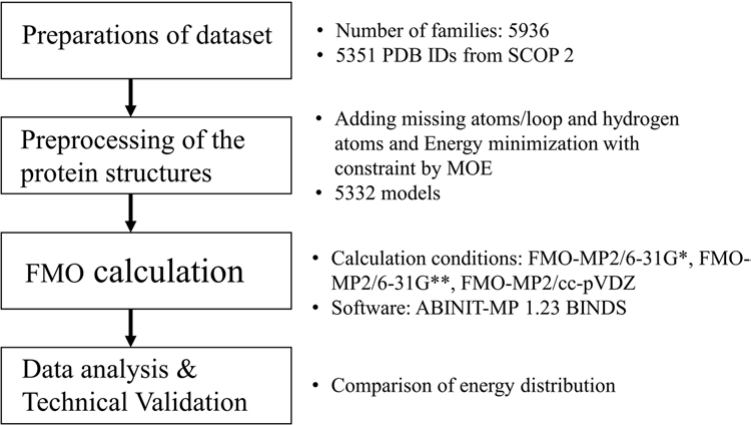
Flowchart of this study.

A flowchart of this study is shown in Fig. 2. First, data for the target proteins were obtained from SCOP2, and model structures for FMO calculations were created from these protein structures. Finally, the data from FMO calculations were analyzed.

### Preparation of dataset

The latest structure list (29 June 2022) was retrieved from the SCOP2 website. The list contains 36,900 structure information items such as the classification of SCOP and the corresponding PDB ID. Each family may contain multiple PDB IDs. In such cases, the first PDB ID from the list was selected, resulting in 5,936 PDB IDs. In addition, multiple domains were assigned to a family using a single combination of PDB and chain IDs. For example, in the case of “1aaa A:1–100… 1aaa A:101–200”, two different families were selected from one PDB ID and its chain combination. In such cases, FMO calculations were performed on all residues within the chain ID to prevent exposure of the hydrophobic core of the protein and to ensure the accurate calculation of the total energy for each chain. Consequently, the number of unique PDB ID and chain ID combinations was reduced to 5,351, which were subjected to FMO calculations.

### Preprocessing of the protein structures

The structures employed as input files for the FMO calculations must be chemically valid. Given that X-ray crystal structures typically lack hydrogen atoms and that some residues may have missing atoms, it is imperative to construct model structures suitable for FMO calculations while preserving as much experimental information as possible. Automation is essential to facilitate high-throughput calculations. The methodology employed in this study was developed with reference to the procedures utilized in the construction of the FMODB^39^. To facilitate FMO calculations and subsequent analyses, all non-natural amino acids were converted to their corresponding natural amino acid counterparts using the functionalities provided by MOE (Molecular Operating Environment)^40^. For residues for which the initial conversion was unsuccessful, a correspondence table provided by the PDB was used to guide the transformation. In cases where atoms or entire residues were missing, a homology model with 100% sequence identity and complete atomic information was generated using the homology modeling function implemented in MOE. The missing parts were then transplanted into the experimental structure by superposition based on the coordinates of the surrounding residues. Energy minimization calculations were conducted on all model structures, irrespective of whether transplantation was necessary, using the Amber10:EHT force field implemented in MOE with constraints applied to the initial positions. Hydrogen atoms were generated using the Protonate3D module. Residues containing transplanted atoms were subjected to positional restraints with a tether value of 1.0, whereas all other non-hydrogen atoms were restrained with a tether value of 0.5 using the MOE parameters, where smaller tether values correspond to stronger constraints on the initial positions. Hydrogen atoms were not constrained because they were added during model construction and were not present in the experimental structures. In this study, metal ions, water molecules, and ligand molecules were excluded from the model structures because the primary objective was to provide FMO calculation data for fundamental protein folds.

Despite these procedures, some structures remain difficult to model. Manual model building was attempted to maximize the amount of FMO calculation data. Initially, we attempted to build structures by utilizing the Structure Preparation module of MOE. When this was unsuccessful, model structures were obtained from AlphaFold DB^6^ or ColabFold^41^. Finally, 5,332 structures, representing 99.6% of the total, were successfully modeled and subjected to FMO calculations. The remaining 19 structures were listed in Table 2.

**Table 2:** List of PDB IDs for which FMO calculation structures could not be obtained due to modeling errors.

PDB ID and chain ID
1di1_A, 1jmu_A-B, 1uc9_A, 2ex3_J, 2g6t_A, 2gnx_A, 2im9_A, 2pva_A, 3a1j_A, 3dh4_C, 3if8_A-B, 3j8c_E, 3opb_A, 4bq6_C-D, 4dgw_C, 4uy4_A, 4yg8_A, 5byh_M, 5d6s_E

### FMO calculations and data analysis

The FMO calculations were performed using SQUID (Supercomputer for Quest to Unsolved Interdisciplinary Data Science, http://www.hpc.cmc.osaka-u.ac.jp/en/squid/), a supercomputer consisting of a group of CPU nodes, a group of GPU nodes, and a group of vector nodes, developed and operated by Osaka University. In this study, only the CPU-node group was used. The configuration is as follows. The CPU node group has 1,520 nodes, each of which has two processors (Intel Xeon Platinum 8368 (Icelake)) with a clock speed of 2.40 GHz and 38 cores (76 cores in total per node). The main memory capacity is 256 GB. A Mellanox InfiniBand HDR (200 Gbps) is used for inter-node connectivity. The theoretical computing performance of the SQUID’s CPU nodes was 8.871 PFLOPS. A Python script was developed to extract IFIE and PIEDA data from the log files generated by the FMO calculations. The extracted data were subsequently converted into tabular format and visualized using the Matplotlib library to illustrate trends in the energy distributions. All FMO calculations were performed using our own customized version of ABINIT-MP version 1.23. The convergence rates of the FMO calculations are summarized in Table 3. IFIE and PIEDA values were successfully obtained for 99% or more of the calculated structures. Only fragment pairs without the dimer-ES approximation^42,43^ were analyzed for the distribution of IFIE and PIEDA.

**Table 3:** Convergence rates of FMO-MP2/6-31 G*, FMO-MP2/6-31 G**, and FMO-MP2/cc-pVDZ.

Calculation condition	# of FMO data	Total	Convergence rate (%)	PDB IDs for which FMO calculation failed
FMO-MP2/6-31 G*	5313	5332	99.6	1h71_P,1k8w_A,1ml9_A,1nlt_A,1ppj_F, 1tex_A,1xm7_A,1z0s_A,1z8g_A,2b9d_A, 2h3o_A,2xdj_F,3hna_A,3mtv_A,3x2r_B, 4kh9_A,4o3m_A,5fig_C,5lye_A
FMO-MP2/6-31 G**	5311	5332	99.6	1h71_P,1k8w_A,1ml9_A,1nlt_A,1tex_A, 1xm7_A,1z0s_A,1z8g_A,2b9d_A,2h3o_A, 2vz8_A,2xdj_F,3mtv_A,3x2r_B,4egc_A, 4kh9_A,4o3m_A,4o9x_A,5amr_A,5fig_C,5lye_A
FMO-MP2/cc-pVDZ	5307	5332	99.5	1h71_P,1k8w_A,1ml9_A,1nlt_A,1s7e_A,1tex_A, 1xm7_A,1z0s_A,1z8g_A,2b9d_A,2h3o_A, 2o3o_B,2vz8_A,2xdj_F,3m63_A,3mtv_A, 3x2r_B,4egc_A,4kh9_A,4lp7_C,4o3m_A, 4o9x_A,5amr_A,5fig_C, 5lye_A

## Data Records

This dataset named FMO-SCOP-29Jun2022^44^ provides protein structural data and the corresponding results of the FMO calculations (Fig. 1c). The input structures for the FMO calculations are provided in PDB format. These structures are modified models that include added hydrogen atoms and complementary residues to ensure convergence of the FMO calculations. The FMO calculations using the MP2/6-31 G*, MP2/6-31 G**, and MP2/cc-pVDZ results included IFIE and PIEDA, which were calculated per fragment based on amino acid residues. As is well established among researchers utilizing the FMO method, it is crucial to note that the default fragmentation was performed at sp3 bonds rather than at typical peptide bonds in the main chain. The data are provided in a simple tabular format in plain text files. Each row of the dataset comprises the PDB ID, residue name, residue number, inter-fragment distance, and IFIE and PIEDA values. The three TSV files corresponding to the FMO-MP2/6-31 G*, FMO-MP2/6-31 G**, and FMO-MP2/cc-pVDZ levels of theory contain 228,158,975, 222,506,834, and 221,978,084 IFIE records, respectively. The number of fragment pairs for which the dimer-ES approximation was not applied (i.e., rows where the value in the “approx” column is “F”) and thus PIEDA energies are available, is 7,856,291, 7,814,304, and 7,804,181 for FMO-MP2/6-31 G*, FMO-MP2/6-31 G**, and FMO-MP2/cc-pVDZ, respectively. The data for the mulliken charge was also added for each calculation condition. The total size of the dataset is approximately 6.7 GB after compression, and made available under a Creative Commons Attribution (CC-BY) license from figshare^44^.

## Technical Validation

### Strategy of technical validation

The dataset used in this study was generated by performing FMO calculations on model structures using three levels of theory: FMO-MP2/6-31 G*, FMO-MP2/6-31 G**, and FMO-MP2/cc-pVDZ. Next, we compared the distribution of the inter-fragment energies calculated by each method with the characteristics of the interactions that the combination can consider to verify that IFIE or PIEDA are expressed as the intended values for the basis sets.

### Validation of distribution of IFIE and PIEDA

**Fig. 3:**
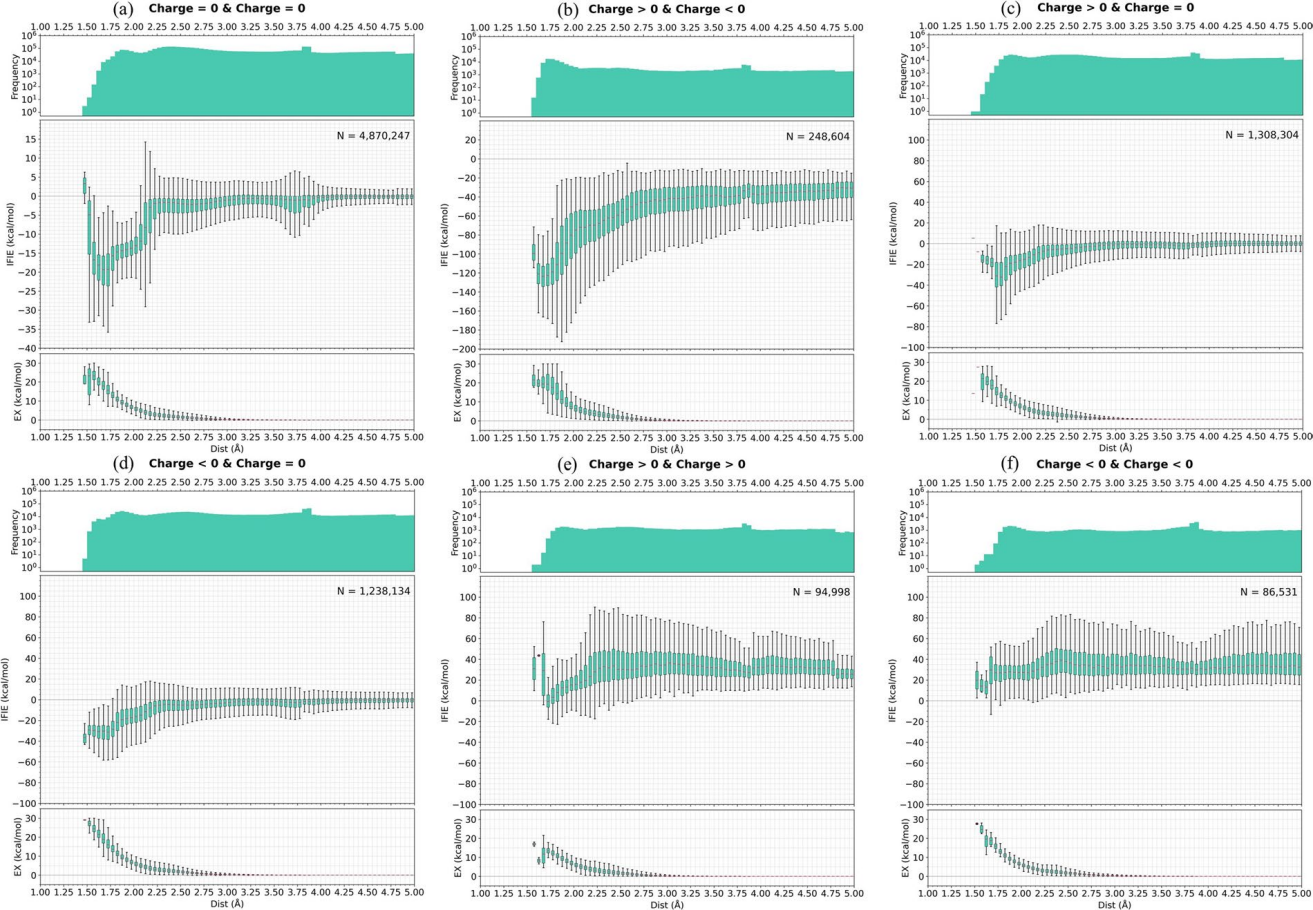
IFIE and EX energy component (from PIEDA) values calculated with FMO-MP2/6-31 G* for each inter-fragment distance bin, with an EX energy threshold of 30 or less. The upper distributions indicate the number of fragment pairs within each distance range. (**a**) neutral fragment pairs and (**b**) attractive charged fragment pairs. (**c**) Fragment pairs with one positively charged fragment and another neutral. (**d**) Fragment pairs with one negatively charged fragment and another neutral. (**e**) Positively and (**f**) negatively charged fragment pairs.

Takaya *et al*. analyzed the distribution of IFIE values for each distance in the FMODB registration structure and reported that it showed a distribution similar to a Morse potential^25^. In this study, we conducted a similar analysis, and although the datasets were different, they showed the same trend. In addition, in FMO calculations targeting proteins, the formal charges of fragments derived from amino acid residues can assume values other than zero. This means that the scale of the IFIE value can differ significantly even for the same amino acid residue. Therefore, the data was divided into charge combinations for each fragment pair, where the inter-fragment distance was defined as the shortest interatomic distance between two fragments, including hydrogen atoms. The distributions of pairs consisting of two neutral fragments and two attractive charged fragments (i.e., the combination of formal charges is 1 or more and −1 or less in a fragment pair) are shown in Fig. 3a and b, respectively. In a previous study^25^, the interactions of neutral fragment pairs and ion pairs were analyzed using hydrogen bond interaction data. For the FMO-MP2/6-31 G* dataset, both distributions were generally consistent with those previously reported despite differences in the distance definition and protein dataset. Other charge combinations of the fragment pairs are summarized in Fig. 3c–f.

**Fig. 4:**
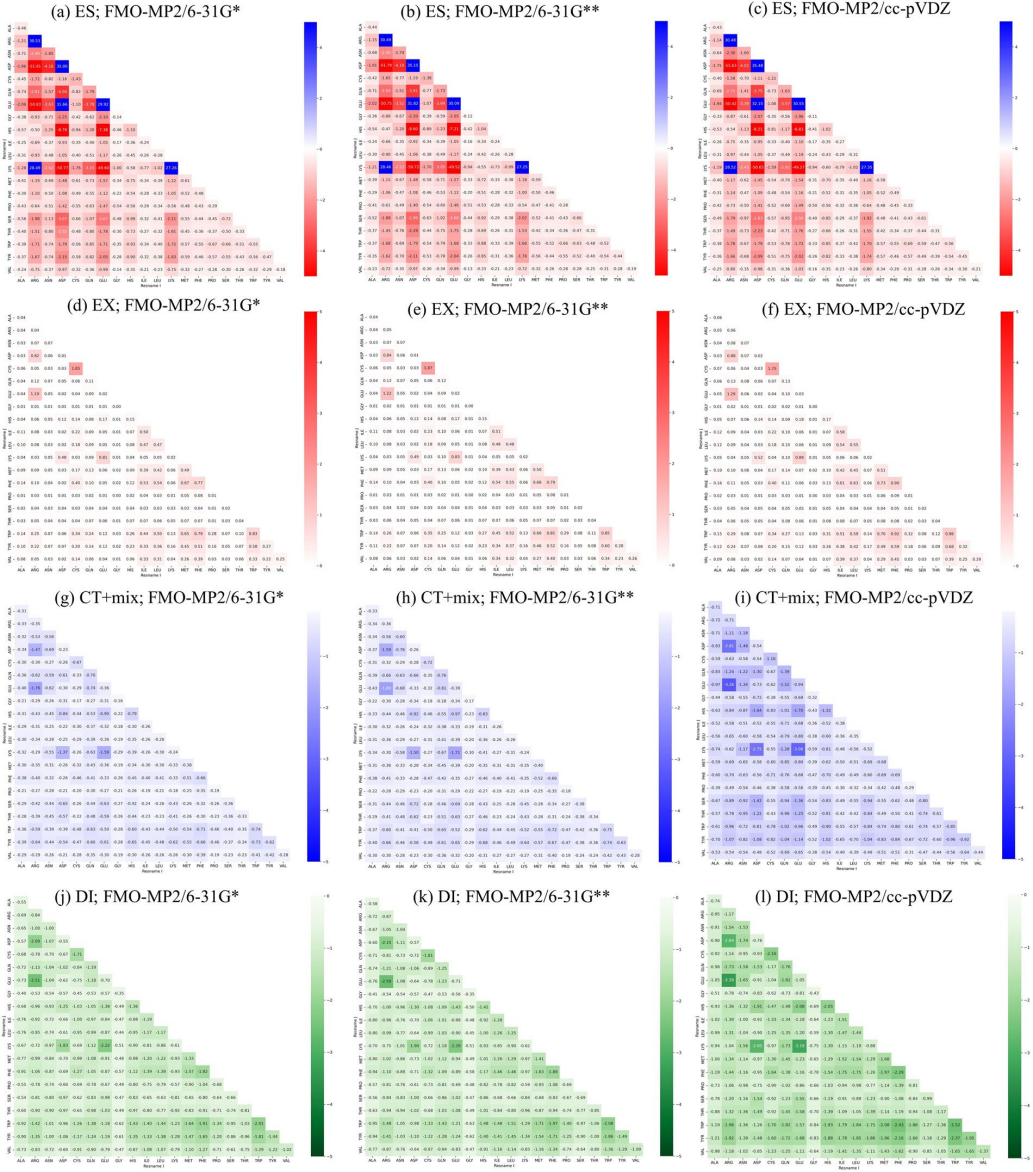
Heatmaps of the median ES, EX, CT + mix, and DI values calculated for all possible pairs of 20 amino acid residues using the following FMO calculation conditions: FMO-MP2/6-31 G*, FMO-MP2/6-31 G**, and FMO-MP2/cc-pVDZ. Subfigures (**a**–**c**), (**d**–**f**), (**g**–**i**), and (**j**–**l**) show ES, EX, CT + mix, and DI, respectively.

This dataset also provides fragment-based IFIE and PIEDA values. We verified that the calculated energies exhibited the expected characteristics of the MP2 method. In QM calculations, the choice of method and basis set determines the range of electron behaviors that is considered. The MP2 method accounts for electron-electron interactions in multi-electron systems, enabling an accurate evaluation of CH/π and π–π interactions based on dispersion forces. Given the presence of π electrons in double bonds and aromatic rings, the MP2 method is particularly suitable for proteins containing aromatic amino acids such as Tyr, Trp, and Phe. The 6-31 G* basis set includes polarization functions for non-hydrogen atoms but not for hydrogen atoms. No exhaustive analyses have been conducted on the differences between different levels of theory. Therefore, we compared the FMO-MP2/6-31 G*, FMO-MP2/6-31 G**, and FMO-MP2/cc-pVDZ PIEDA components, which were calculated for fragments with the same coordinates. Heatmaps of the median ES, EX, CT+mix, and DI values calculated for all possible pairs of 20 amino acid residues using the FMO calculation conditions are shown in Fig. 4. The maximum and minimum ratios of the median value of each PIEDA component for each basis set combination, as well as the associated amino acid pairs, are summarized in Table 4. In this analysis, pairs of Cys fragments that form disulfide bonds are excluded.

**Table 4:** Maximum and minimum ratios of basis set 1 to 2 for the median values of the PIEDA components and their amino acid pairs.

Basis set 1	Basis set 2	PIEDA	MAX ratio	MIN ratio	MAX pair		MIN pair	
FMO-MP2/6-31 G**	FMO-MP2/6-31 G*	ES	1.03	0.84	Asp	Cys	Gly	Gly
		EX	1.14	1.00	Arg	Gly	Ala	Asn
		CT + mix	1.12	0.98	Asp	Asp	Pro	Pro
		DI	1.07	0.97	Ile	Ile	Gly	Pro
FMO-MP2/cc-pVDZ	FMO-MP/6-31 G*	ES	1.15	0.78	Arg	Pro	Gly	Gly
		EX	1.50	0.95	Ala	Gly	Cys	Cys
		CT + mix	2.61	1.06	Glu	Glu	Phe	Phe
		DI	1.62	1.21	Asn	Asp	Pro	Pro
FMO-MP2/cc-pVDZ	FMO-MP2/6-31 G**	ES	1.21	0.87	Arg	Pro	Cys	Cys
		EX	1.50	0.94	Gly	Gly	Cys	Cys
		CT + mix	2.40	1.04	Glu	Glu	Phe	Phe
		DI	1.56	1.15	Asn	Asp	Leu	Leu

All values are ratios based on the numerical data in Fig. 4, compared using “basis set 2” as the denominator.

The median ES, EX, CT+mix, and DI energies for the FMO-MP2/6-31 G* and FMO-MP2/6-31 G** datasets are almost identical, with a maximum ratio of approximately 1.14 for EX and a minimum ratio of approximately 0.84 for ES. Although the 6-31 G** basis set has a larger number of atomic orbitals owing to the inclusion of polarization functions for hydrogen atoms, which could lead to changes in accounting for dispersion forces, only 1.37% of the fragment pairs had an absolute IFIE difference of 1 kcal/mol or more. When IFIE is used as a criterion for selecting important interactions, such as an absolute IFIE difference of 3 kcal/mol or more^45^, the IFIE difference due to the basis set may affect the detection of such important interactions. It is worth noting that all of the hydrogen atoms in the model structures were generated by modeling and optimized using molecular mechanics (Amber10:EHT, implemented in MOE). If FMO-MP2/6-31 G** is used to accurately evaluate the contribution of hydrogen atoms, it may be necessary to optimize the hydrogen atom positions using calculation conditions equivalent to those of MP2/6-31 G**.

**Fig. 5:**
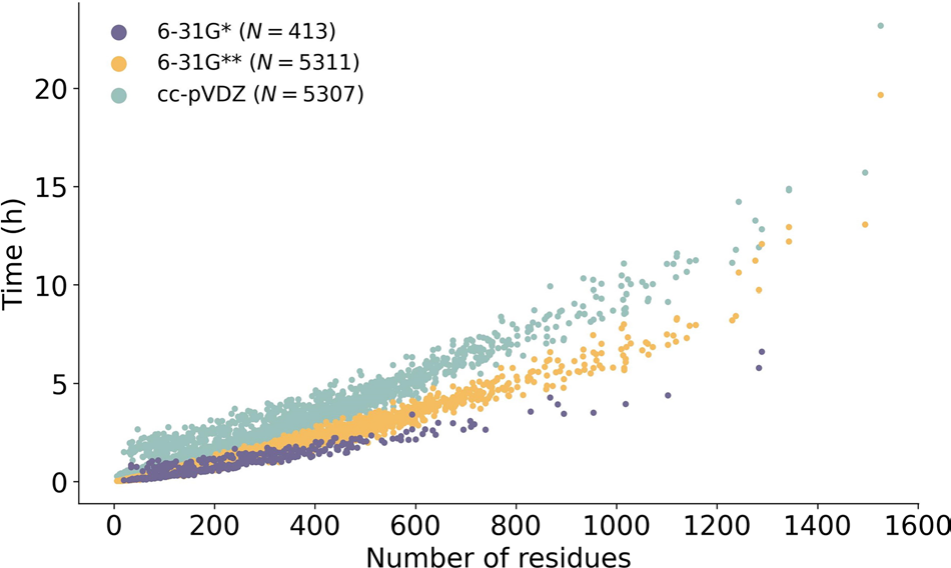
Approximate calculation time (hour) versus the number of residues for different levels of theory. The plot encompasses data obtained using both SMP and MPI parallelization techniques. Only calculation times that used a near-maximum and evenly distributed number of cores across nodes were included. The excluded data mainly consisted of calculations that used an uneven number of cores across nodes and/or only a few cores (e.g., only 4 of 76 cores in a node).

For the analysis of amino acid interactions in proteins, it is reasonable to select FMO-MP2/6-31 G*, which is already the convention. Although the FMO-MP2/6-31 G* calculations were faster than FMO-MP2/6-31 G** calculations (at most 2.0 times, as shown in Fig. 5), the difference in computational time may not be a significant factor in the application of a few target proteins, making the use of both methods a viable option.

Compared to FMO-MP2/6-31 G*, FMO-MP2/cc-pVDZ exhibited larger differences than 6-31 G**. Although the median ES and EX energies were comparable for the FMO-MP2/6-31 G* and FMO-MP2/cc-pVDZ datasets, the median CT+mix and DI energies were more stable in the FMO-MP2/cc-pVDZ dataset (up to approximately 2.6 times and at least approximately 1.62 times, respectively). This is also evident from the shading of the CT+mix and DI heatmaps (Fig. 4g–l), where the FMO-MP2/cc-pVDZ dataset is clearly darker than the other two datasets. As shown in Table 1, the main differences between the cc-pVDZ and 6–31 G* basis sets are their polarization functions and correlation consistency. Furthermore, 34.3% of the fragment pairs had an absolute IFIE difference of 1 kcal/mol or more. The cc-pVDZ basis set incorporates polarization functions for hydrogen atoms and correlation consistency compared to the 6-31 G* basis set, which significantly improved the quality of the wave function. Although the energy values obtained with FMO-MP2/cc-pVDZ were more stable than those obtained with FMO-MP2/6-31 G*, further research is needed to correlate these results with experimental data, such as protein–protein and antibody–antigen binding affinities, to determine whether FMO-MP2/cc-pVDZ is more suitable for explaining biological phenomena or should be used in conjunction with FMO-MP2/6-31 G*.

## Data Availability

ABINIT-MP version 1.23 is available in binary format by following the instructions at https://www.cenav.org/abinit-mp-open_ver-1-rev-22/. MOE 2022.02 is a molecular modeling software package developed and distributed by the Chemical Computing Group (CCG; https://www.chemcomp.com).
